# Ethnic Differences in and Childhood Influences on Early Adult Pulse Wave Velocity: The Determinants of Adolescent, Now Young Adult, Social Wellbeing, and Health Longitudinal Study

**DOI:** 10.1161/HYPERTENSIONAHA.115.07079

**Published:** 2016-05-11

**Authors:** J. Kennedy Cruickshank, Maria J. Silva, Oarabile R. Molaodi, Zinat E. Enayat, Aidan Cassidy, Alexis Karamanos, Ursula M. Read, Luca Faconti, Philippa Dall, Ben Stansfield, Seeromanie Harding

**Affiliations:** From the Cardiovascular Medicine Group, Division of Diabetes and Nutritional Sciences, Kings College London, London, United Kingdom (J.K.C., M.J.S., Z.E.E., A.K., L.F., S.H.); Medical Research Council/Chief Scientist’s Office (MRC/CSO), Social and Public Health Sciences Unit, Institute of Health and Wellbeing, University of Glasgow, Glasgow, Scotland, United Kingdom (O.R.M., A.C., U.M.R.); and Institute for Applied Health Research, Glasgow Caledonian University, Glasgow, Scotland, United Kingdom (P.D., B.S.).

**Keywords:** adult, blood pressure, ethnicity, longitudinal studies, pulse wave velocity

## Abstract

Early determinants of aortic stiffness as pulse wave velocity are poorly understood. We tested how factors measured twice previously in childhood in a multiethnic cohort study, particularly body mass, blood pressure, and objectively assessed physical activity affected aortic stiffness in young adults. Of 6643 London children, aged 11 to 13 years, from 51 schools in samples stratified by 6 ethnic groups with different cardiovascular risk, 4785 (72%) were seen again at aged 14 to 16 years. In 2013, 666 (97% of invited) took part in a young adult (21–23 years) pilot follow-up. With psychosocial and anthropometric measures, aortic stiffness and blood pressure were recorded via an upper arm calibrated Arteriograph device. In a subsample (n=334), physical activity was measured >5 days via the ActivPal. Unadjusted pulse wave velocities in black Caribbean and white UK young men were similar (mean±SD 7.9±0.3 versus 7.6±0.4 m/s) and lower in other groups at similar systolic pressures (120 mm Hg) and body mass (24.6 kg/m^2^). In fully adjusted regression models, independent of pressure effects, black Caribbean (higher body mass/waists), black African, and Indian young women had lower stiffness (by 0.5–0.8; 95% confidence interval, 0.1–1.1 m/s) than did white British women (6.9±0.2 m/s). Values were separately increased by age, pressure, powerful impacts from waist/height, time spent sedentary, and a reported racism effect (+0.3 m/s). Time walking at >100 steps/min was associated with reduced stiffness (*P*<0.01). Effects of childhood waist/hip were detected. By young adulthood, increased waist/height ratios, lower physical activity, blood pressure, and psychosocial variables (eg, perceived racism) independently increase arterial stiffness, effects likely to increase with age.

## Abstract 2

Supplemental Digital Content is available in the text.

Arterial stiffness, measured as aortic pulse wave velocity (PWV), has become a major intermediary outcome for cardiovascular events and mortality. Two recent meta-analyses show increasing PWV’s powerful predictive ability, independent of ambient blood pressure (BP), and other risk factors.^1,2^ PWV may therefore become an appropriate target for interventions. However, very few studies have examined its development over time, particularly in young adults. As arterial stiffening develops over the life course, detecting factors that determine its progression should be useful in delaying or preventing its decline. Measurement of PWV by several techniques, whether over the so-called carotid-femoral pathway or estimated over the more central aorta, seems to be repeatable and less variable than BP.^3,4^ An earlier cross-sectional meta-analysis suggested only BP and age to be major influences, with a minor role for other standard risk factors.^5^ Longitudinal data were not considered.

Studies in people of different ethnic groups at varying vascular risk in the same setting are rare although our earlier study with long-term follow-up showed its utility in black Caribbean and Indian origin people in the United Kingdom.^6^ Here, we tested how factors measured twice previously in childhood in the Medical Research Council’s Determinants of Adolescent, Now Young Adult, Social Wellbeing and Health (DASH) study, particularly components of body mass and BP,^7–10^ affected the emergence of PWV in young adults. We tested the hypothesis that increased earlier childhood BP and fat mass, as well as current BP and body mass index (BMI), would be the main determinants of PWV across all ethnic groups.

## Methods

Design details of the of the DASH study can be found at http://dash.sphsu.mrc.ac.uk and in a published cohort profile.^7^ The DASH sample was recruited between 2002 and 2003, from 51 schools in 10 London boroughs. A total of 6643 students, aged 11 to 13 years, stratified in samples of ≈1000 children in each of 6 ethnic groups, as below, in first and second years of secondary school, took part in the baseline survey. In 2005 to 2006, 4785 (88% of children in 49 schools, 72% of the cohort), aged 14 to 16 years, took part in the first follow-up. In 2013, when the cohort was aged 21 to 23 years, 81% of the baseline sample was located. A subsample of 665 (97% of participants invited) took part in a 10% pilot follow-up study, which was completed in March 2014. Response rates (≥90% of the invited pilot sample) were similar by ethnicity and sex. The pilot sample consisted of 107 white British, 98 Indian, 111 Pakistani or Bangladeshi, 132 black African (mainly Nigerian and Ghanaian), 102 black Caribbean, and 115 other ethnicity, chosen to give a representative spread by sex and socioeconomic circumstances (SEC) across all 10 London boroughs and 51 schools. The aim was to reflect the diversity of London’s multicultural population, so as to detect psychosocial and physical factors that might influence the populations’ varying risk of different health outcomes.

Approval for the study was obtained from the National Health Service Research Ethics Committees. Written informed consent was obtained from participants. Ethnicity in DASH was measured by self-reported ethnicity, checked against reported parental ethnicity and grandparents’ country of birth. For analysis, Bangladeshis and Pakistanis were combined because of the relatively small sample size of the Bangladeshi. Both groups are distinctly different from Indians having been more economically disadvantaged and predominantly Muslim.

### Physical Measures

Measurement protocols can be found at the Web link above. Nurses and research assistants were trained for 1 week before the start of fieldwork and were recertified at 6 monthly intervals. All equipment was checked and calibrated regularly by the field supervisors. In adolescence, all assessments were conducted in schools >2 to 3 days per school. At 21 to 23 years, participants were given a choice of locations and assessments took ≈2 hours. Locations included their local general practitioners’ surgeries, local community pharmacies, Clinical Research Centres, and King’s College London (from where the study was run).

Protocols for anthropometric measurements were taken from the World Health Organization manual. Height and sitting height were measured using portable Leicester stadiometers (Seca) and standard height stools, and weight using Salter electronic (11–13 years) and Tanita HD-352 scales (14–16 and 21–23 years). Systolic BP and diastolic BP were measured using validated OMRON M5-I semiautomatic devices and appropriately sized cuffs, after the participant had sat quietly for a timed 5 minutes, with >1 minute between 3 subsequent readings. The mean of the second and third readings was used in analysis, as previously reported.^8,10^ Ambient air temperature was recorded with a digital thermometer. Bioimpedance was measured using the Tanita HD-352 scales (14–16 years) and the Bodystat 1500MDD (21–23 years). At 21 to 23 years, PWV and brachial BP were also measured using the Arteriograph 24-hour device, previously calibrated and standardized.^4^ The device records ≤8 cardiac cycles, 3 separate times in 1 sitting. The aortic path length is measured with a long-arm caliper, from suprasternal notch to pubic rami. After the readings, a blood sample of 25 mL was taken.

Physical activity (PA) was not measured in detail during adolescence. In the follow-up study, a subsample of participants n=334, 76% of those invited, wore a waterproofed ActivPAL monitor continuously for 5 days. Worn on the front of the thigh, the monitor is valid for identifying sitting standing and walking.^11^ The following were derived and reported per day: steps taken, upright time, time walking at >100 steps/min (equivalent to moderate-vigorous PA), sit-to-stand transitions, and proportion of daytime sitting (between 09:00 and 21:00) spent in prolonged (>20 minutes) bouts.

### Social Measures

A self-administered questionnaire measured other social factors, including health behaviors, racism, and SEC. Reported racism was assessed using standardized questions on unfair treatment on the grounds of race, skin color, country of birth, or religion in various locations (school, street, work, etc).^12^ In adolescence, SEC was measured through parental employment plus the Family Affluence Scale based on number of cars, computers, holidays, etc.^13^ In adulthood, SEC was measured through own education and employment.

### Statistical Method

**Figure 1.**
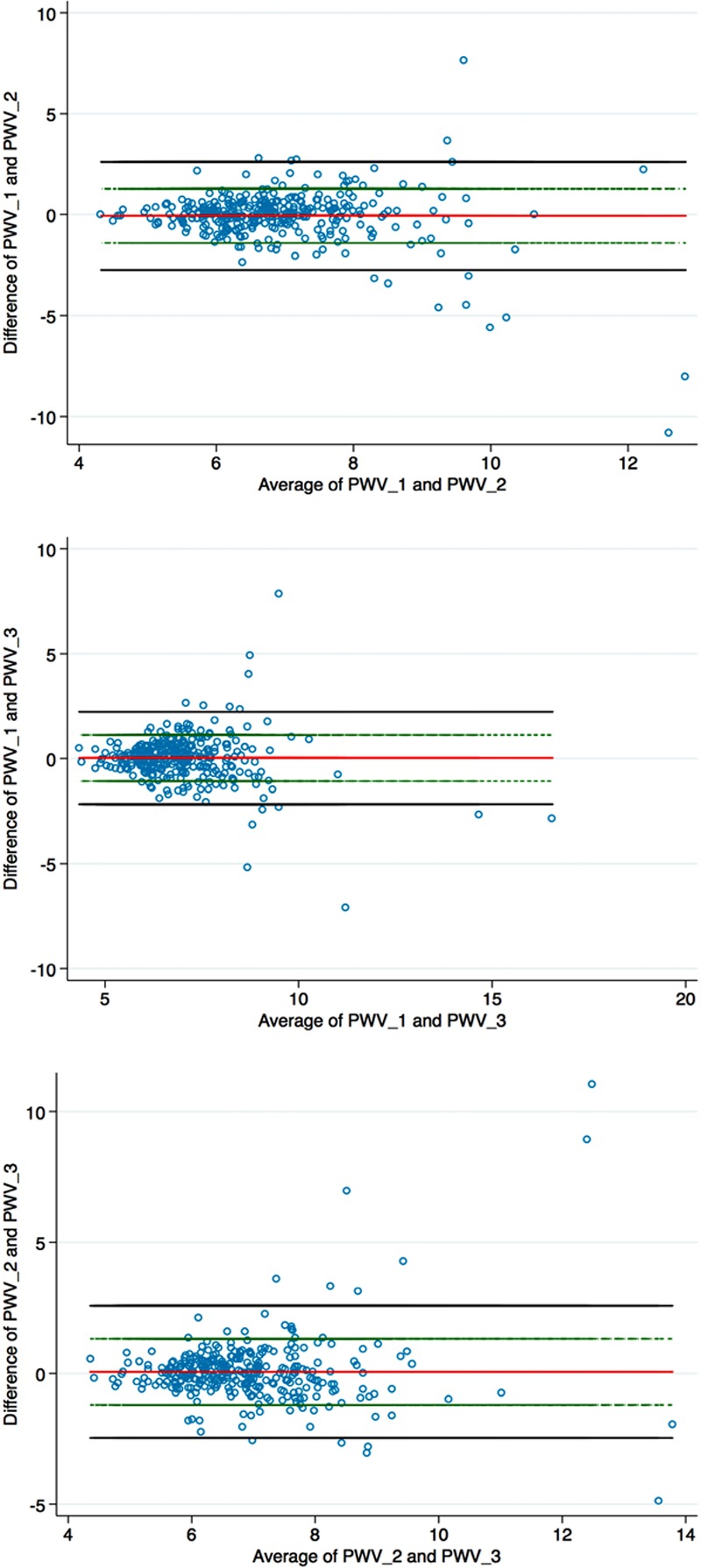
Reproducibility of pulse wave velocity (PWV) in the first 3 measurements: Bland–Altman plots. These are differences between successive PWV measures (y axis) plotted against the mean of those values (x axis).

Bland–Altman plots showed that almost all observations were within the limits of agreement and the bias was close to zero (Figure 1). Therefore, we used the mean of the 3 separate PWV measurements for each participant. We also used the mean brachial BP from the Arteriograph, from the first 3 measures taken with PWV.

#### Model Building Approach

The core model contained age (at 21–23 years, yearly), brachial BP (mean BP in separate analyses) and ethnicity. We first examined the influence of current exposures at 21 to 23 years—different body size measures (BMI, fat mass indexed to height, fat-free mass (waist/height), social exposures (PA, smoking, racism, SEC). We then tested the influence of similarly measured exposures at ages 11 to 13 years and 14 to 16 years, by adding to the model with current exposures so that the final model gave insight into current exposures adjusted for the exposures in adolescence, and for any independent effects of exposures at the 3 age per time points. Each variable was tested in univariate models (added to the core model) before final multivariable linear regressions were conducted. The final models fitted depended on comparisons of the same models (quadratic with linear) tested with the likelihood-ratio test and Bayesian Information Criteria. Software R 3.1.2 was used to plot the frequency curves and Bland–Altman graphs with all the modeling performed using Stata 13. Given the small sample size, potential statistical significance was considered at *P*<0.10.

## Results

By age 21 to 23 years, 45% of men but 59% of the women had higher education, and 58% and 53% were employed (Table 1). A high proportion, 48%, of men smoked, as did 36% of women. With growth completed, young men averaged 13 cm taller than the women having been the same height aged 11 to 13 years. Although male BMIs were marginally lower, their fat mass indexed to height was nearly half that in the women, despite significantly larger waists. Resulting systolic BPs were also substantially higher in the men but diastolic BPs were not significantly higher.

**Table 1.**
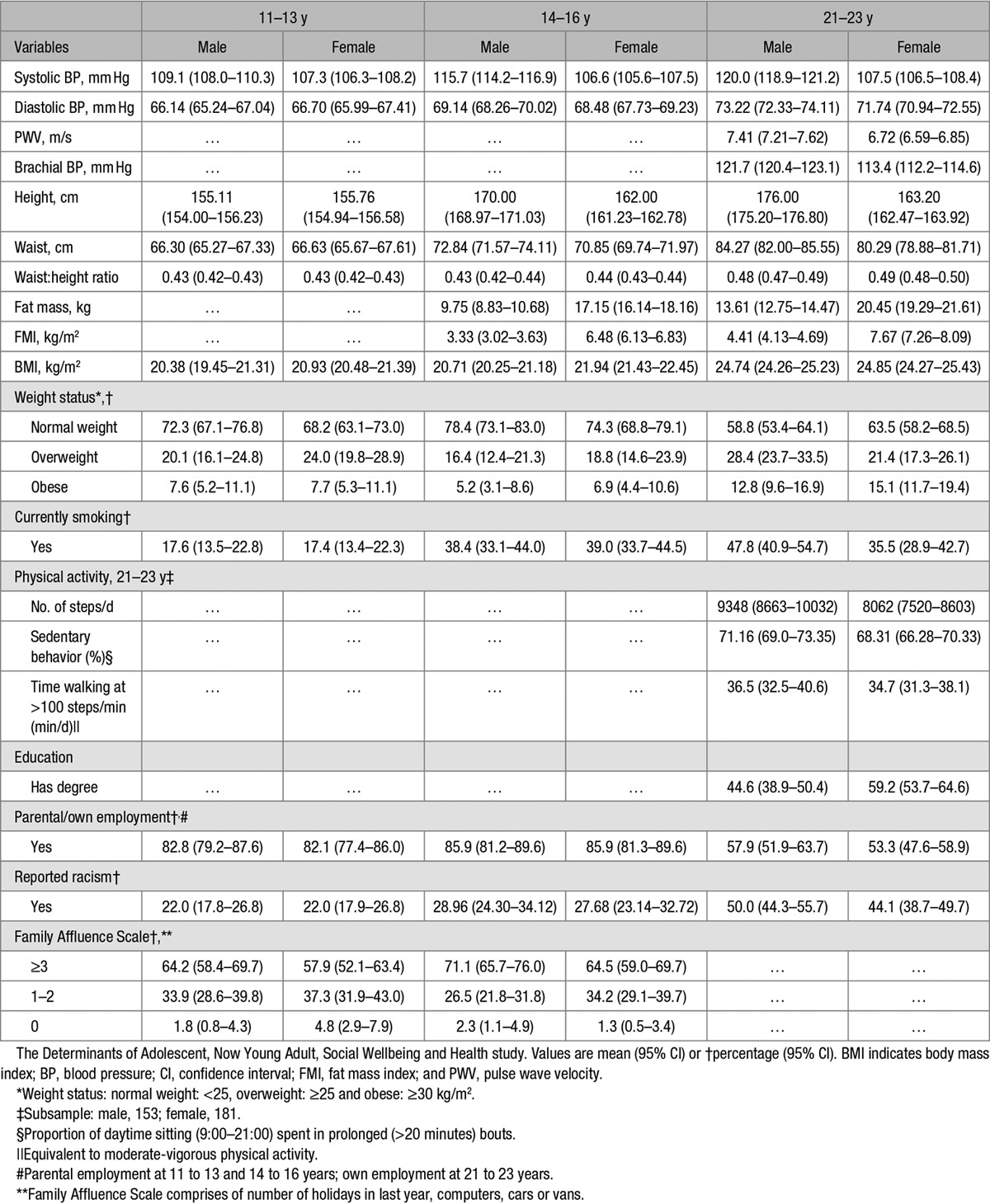
Descriptive Profile of the Sample (95% CI) or Percentage (95% CI) by Sex

**Figure 2.**
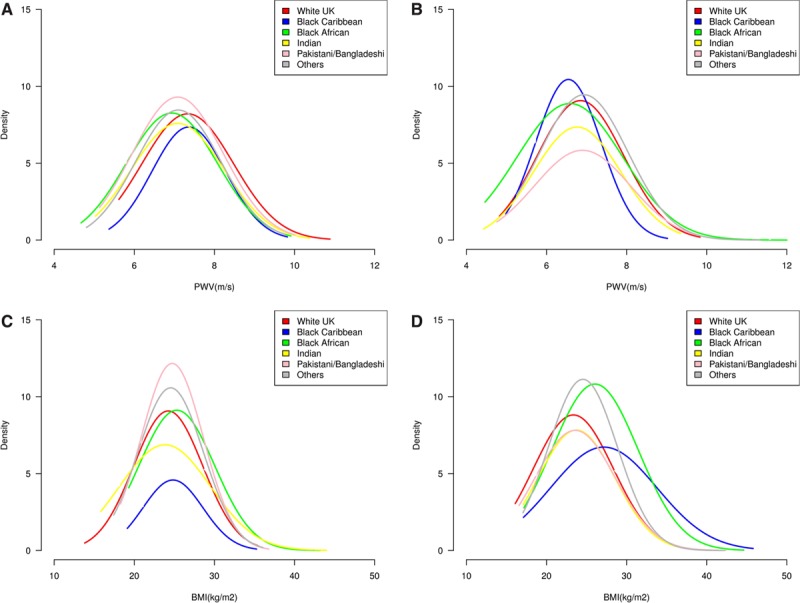
Normal curves as density plots for the distribution of frequencies for pulse wave velocity (**A**) male and (**B**) female and for body mass index (**C**) males and (**D**) females, by ethnicity at 21 to 23 y. The Determinants of Adolescent, Now Young Adult, Social Wellbeing and Health study.

Bland–Altman plots showed excellent repeatability for PWV measures (Figure 1). Mean differences of first to second, second to third or first to third sets of readings (averaging 6–8 cardiac cycles recorded for each) were −0.06, 0.03, and 0.06 m/s, respectively, with 95% confidence intervals of ±2 m/s each. Plots for the distribution of frequencies for BMI and PWV (Figure 2) were close to Gaussian; those for white UK and black Caribbean men were shifted slightly right of others, but markedly left-shifted, reflecting lower mean values, in black Caribbean women, who were larger (Figure 2B).

Unadjusted PWV (Table I in the online-only Data Supplement) was similar in black Caribbean and white UK young men (mean±SD, 7.9±0.3 versus 7.6±0.4 m/s) but lower in other groups at similar systolic BPs (120 mm Hg), BMIs (24.6 kg/m^2^), and waists (84.3 cm). In regression models, black Caribbean women had lower PWVs (6.6±0.1 m/s) despite right-shifted BMIs and waists (Figure 2A; *P*<0.05) than did white UK women (6.9±0.1 m/s). Using waist/height adjustment instead of BMI, which can be misleading for fat:lean mass proportions, the PWV difference was slightly greater at −0.5 (95% confidence interval, −0.95 to −0.06) m/s. However, using fat or lean mass did not alter the results (not shown). Further adjustments for 11- to 13-year waist measures also made impacts, as they did in men.

With BP and age alone, ethnicity had no independent effect but, after waist/height inclusion, did so in (West) African and black Caribbean, with a suggestion in Indian women for lower PWV by some 0.4 to 0.5 m/s, with age and systolic BP still significant. Including current social effects (Table 2, column 3) retained waist/height, which increased PWV 2.75 m/s, now with a racism impact (0.3 m/s) also. Finally, with extra adjustments for all adolescent factors, lower PWV persisted in black African men, with powerful impacts from current larger waists and some from family affluence (at 11–14 years) increasing PWV for all. In those final models, black Caribbean, black African, and Indian women had definitely lower PWV, which was still increased by age, BP, with again a large impact from waist/height, and a racism effect persisting.

**Table 2.**
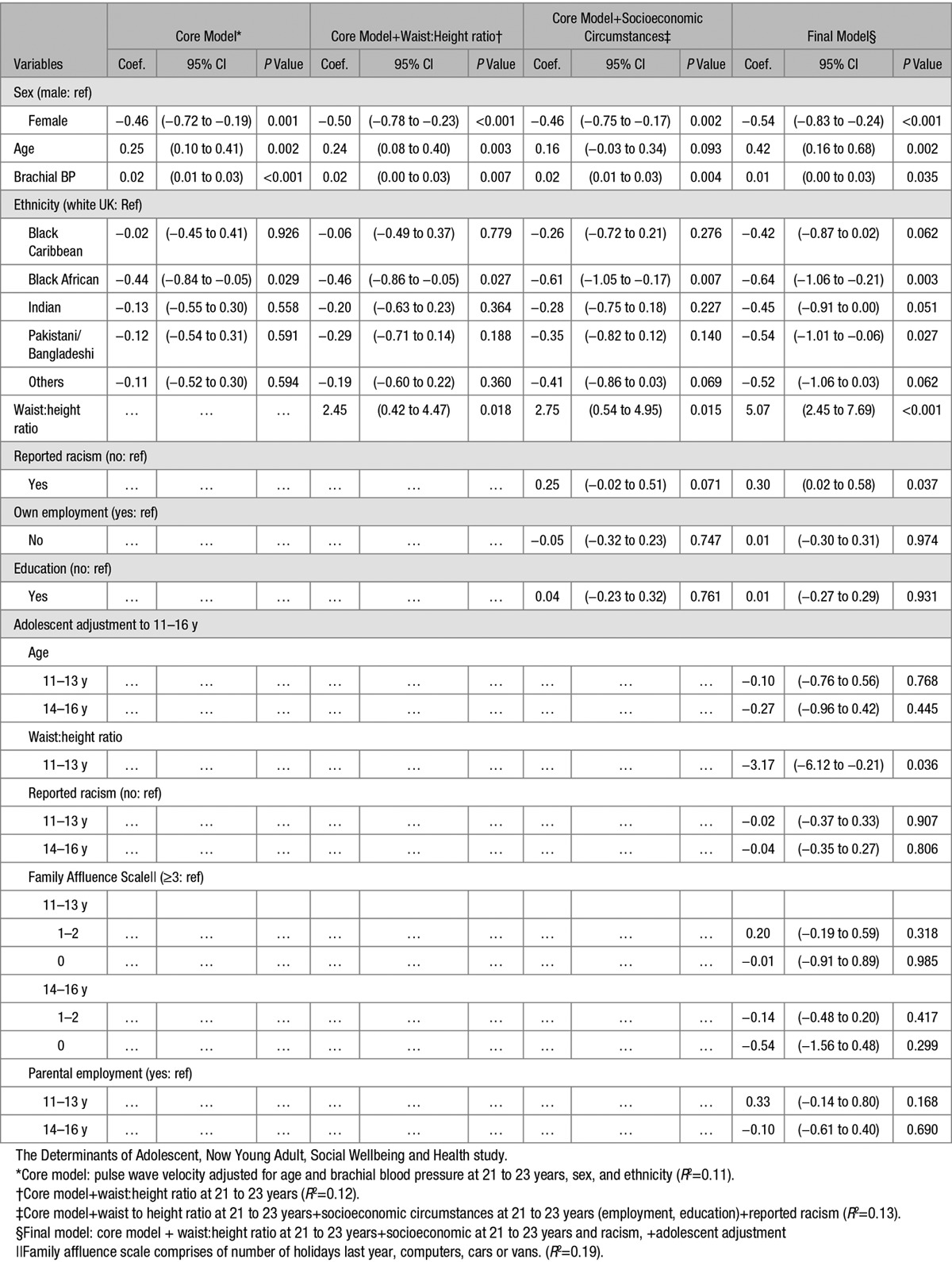
Pulse Wave Velocity at 21 to 23 Years: Influence of Systolic Blood Pressure, Waist:Height Ratio, and Social Exposures From Early Adolescence

A subsample (n=334) had objective PA measures (ActivPal), directly measured >5 days. Both men and women spent some 70% of waking hours sedentary, but overall spent ≈36 minutes/d in moderate to vigorous activity (Table 1). We then assessed whether such PA had any effects on PWV in this smaller sample, independent of the other variables above, (ie, in a restricted model). Its 2 major indices, number of steps/day and particularly time walking at >100 steps/min, were both negatively (ie, protectively) associated with PWV (Table 3). The ethnic effect in black African men and for BP was retained, after full adjustment for other variables. Neither BMI (not shown) nor waist:height ratio at 21 to 23 years had any independent impact.

**Table 3.**
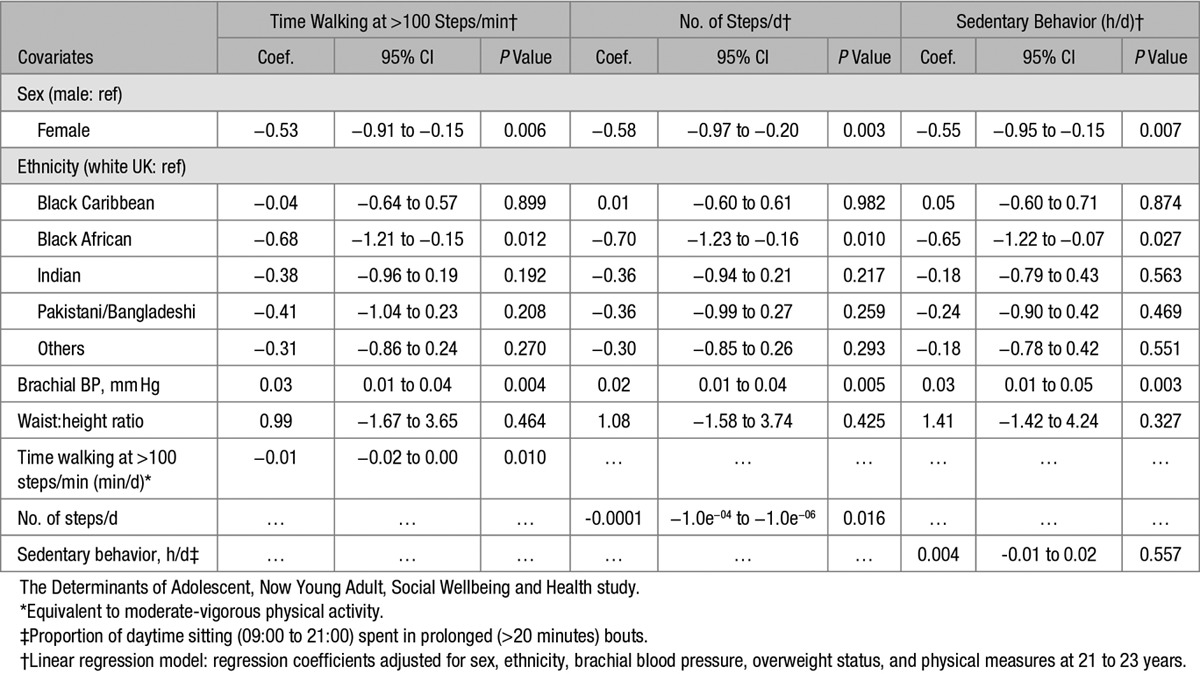
Pulse Wave Velocity at 21 to 23 Years: Influence of Physical Activity at 21 to 23 Years

## Discussion

This initial follow-up at ages 21 to 23 years old has proved highly successful with >97% of those invited attending for visits. Details of recruiting efforts and techniques, allowing choice of place for follow-up, will be published separately. The sampling strategy stratified by the 6 ethnic groups also allowed a reasonable balance by sex among the ≥100 people in each group.

Here, the focus was on assessing determinants of PWV to examine relative impacts of both current anthropometric and BP measures at this latest age in young adulthood and those from the initial 2 full waves of visits at ages 11 to 13 and 14 to 16 years. Adolescent BP incidence data were reported before.^10^ Ethnic differences in BP noted then were no longer evident here, but upward BP changes in directly African and African-Caribbean girls, especially had occurred by the last follow-up, whereas in other groups that rise occurred between 14 to 16 years and 21 to 23 years. Social measures were also included where appropriate in the statistical models.

Within-visit repeatability for PWV measures was excellent and showed no regression to a mean although scatter (variance) tended to increase above ≈9 m/s as the aorta becomes pathologically stiffer.

The regression models determine what factors contribute to PWV, built up progressively from core adjustments for physique just at 21 to 23 years, then including current social then adolescent environment and physique also (Table 2). Perhaps surprisingly, a large independent age effect occurred within this cohort’s 3-year age span in fully adjusted models (0.4, 0.16–0.68 m/s). Arterial aging per se at this age is unlikely, but we suspect an impact from body composition and perhaps change in PA, separate to their direct effects, as below. Any impact of earlier BPs, in either core or full models, was not significant (Table S2, Stata text file output). To our knowledge, no other study has reported the impact of BP measured twice previously in early and late adolescence across ethnic groups. The Avon Longitudinal Study of Parents and Children (ALSPAC) study^14,15^ similarly reported significant negative effects of BMI on PWV but at the age of 10 years, using carotid-femoral PWV, against which the Arteriograph is calibrated^16^ but estimates the more central aortic pathway.^4^ Waist/height ratios, used to estimate intra-abdominal fat, had marked positive effects at 21 to 23 years but, as with BMI in ALSPAC, a minor negative effect from earlier ages. We also tested models examining changes in growth trajectory across the 3 age periods, but neither height alone nor waist/height was significant (Table S2, Stata outputs). Dietary assessments were conducted in a similarly sized and aged Northern Ireland cohort with PWV measured.^17^ Men had higher PWV than women, as generally found until older ages. The slightly higher PWV in white European women than in other ethnic groups (absolute, ie, unadjusted difference only 0.3 m/s) is likely because of their lower BMI, as shown by adjusting.

Intriguingly, data from an older European cohort suggest that BP does not cause the progressive increase in arterial stiffening, measured as PWV, but rather vice versa, with other arterial wall properties also modulating BP change over time.^18^ That study also illustrated the problems of measuring the genuine arterial distance travelled over the carotid-femoral (or rather cardiac-femoral and cardiac-carotid) pathways by PWV in more obese subjects.^19^ That length is less problematic but still relevant for estimates from the Arteriograph with measures taken here by a long-arm caliper, and calibrated against MR-determined aortic length.^4^ Furthermore, recent data from the large Whitehall study^20^ used carotid-femoral PWV at a baseline and 4 years later in longitudinal analyses of ≥60-year olds: 3789 men and 1383 women. Several adiposity indices (general by BMI, central as waist or waist:hip ratio and fat mass by bioimpedance) were independently related at baseline, and from previous screening to change in PWV. Blood C-reactive protein and interleukin-6 as inflammatory markers were also independently related to change in PWV, independently of BP treatment, and other risk factors. That change in direction of how abdominal adiposity is related to PWV, from negative in youth in this study and in ALSPAC to fully positive in Whitehall, strongly suggests fatness around adolescence starts the process, probably of inflammation, that leads to arterial “ageing”.

Results for PA, measured here in the subsample, illustrate again its potentially vital role in maintaining arterial health. During active hours (0900–2100), 70% of both sexes showed prolonged sedentary behavior, defined as multiple bouts >20 minutes with no activity. Periods in moderate-vigorous activity averaged 36 minutes/d.

Several earlier studies using different techniques also reported the impact of even short-term activity at younger ages.^21,22^ Some of these were independent of BP changes, which often were still increased after activity; other studies did not adjust for BP (change). European cohort studies support the modulating role of habitual activity, measured directly as here or by questionnaires, said to be validated for activity measurement. The Young Finns study, using an impedance device to derive PWV and activity by questionnaire, showed >21-year follow-up in an inverse dose–response association in younger and older adults, not adjusted for BP.^23^ The relationship was confirmed with separate measures for carotid stiffness indices, adjusted for systolic BP.^23^ Similar findings were reported from the Danish European youth study.^24^ Activity data here measured >5 days should be representative, even considering that wearing the device might be an incentive to take more exercise. Other possibilities include some genetically predisposed individuals with better ventricular–aortic coupling enjoying PA more. Whether PA in the overweight or obese who seem metabolically healthy, but are still at marked excess cardiovascular risk, improves survival^25^ remains to be tested.

Limitations of the work to date include a relatively small sample size of ≈100 per ethnic group, balanced by sex, but these young people should be representative of the whole cohort of 6000 because a >90% response rate across sampled groups was achieved. Some consider the Arteriograph, as a single-site, cuff-based method to have limitations compared with other devices previously more widely used, despite its calibration by one author’s group (J.K.C.) against MR and other devices.^4^ The exact arterial length over which its PWV values are measured is uncertain (like length measures for all devices except MRI), but it approximates the aortic valve to bifurcation or just below, including less muscular artery than tonometry. Its great advantages are its portability, ease of use, independence of observer, comfort, and acceptability, while well calibrated. The subgroup with objectively measured PA was smaller because of cost issues, so its regression models were more restricted, but again informative across all groups. These issues are being addressed in proposals for further follow-up.

In conclusion, for this initial young adult follow-up of DASH’s multiethnic cohort, PWV was measured with good reproducibility, was lower at given BP levels in young black African, but not in black Caribbean, than in white British men and in all ethnic minority women than in white British women. Current age, BP, waist:height ratios, and a racism effect were still independently related to PWV. From the previous 2 times of measurements conducted in school, the main likely causal factor was a paradoxically protective effect on vessel stiffness from higher waist:height ratio (perhaps allowing excess extravasation of lipid and proteins) at 11 to 13 years. That was followed by a powerful impact worsening PWV of abdominal adiposity (WHtR) at age 21 to 23 years. Effects of earlier BP on PWV at 21 to 23 years were not detected, adding to the growing if still controversial evidence that PWV may as much determine BP as vice versa.^26^

How social variables affect arterial function and perhaps eventual events over time need exploring. Reported racism, perhaps mediated via chronic stress–responses, was more common in women, among those who lived in deprived areas but statistically independent.

### Perspectives

Studies of arterial function, particularly arterial stiffness, measured by PWV in young adults remain scarce, particularly across different ethnic groups whose parents have been at higher cardiovascular risk than host populations. We found that these young adults were generally prepared to attend for demanding mental, physical, and blood testing, having attended twice before at school. Here, current abdominal girth (adjusted for height) was associated with higher PWV, but with an opposite effect earlier aged 11 to 13 years. Objectively measured PA seems to restrain arterial stiffening while apparent responses to racism, especially in women, presumably via stress responses, had direct stiffening effects.

## Acknowledgments

The invaluable support of participants and their parents, the Participant Advisory Group, schools, civic leaders, local GP surgeries and community pharmacies, the Clinical Research Centre at Queen Mary University of London, the Clinical Research Facility at University College Hospital, the survey assistants and nurses involved with data collection, the Primary Care Research Network, and Professor Sanders from the Diabetes and Nutritional Sciences Division at Kings College London for hosting the feasibility study. Seeromanie Harding is the Principal Investigator of DASH. All authors contributed to study design, analyses, and writing of the article.

## Sources of Funding

The study was funded by the MRC (MC_U130015185/MC_UU_12017/13), Chief Scientist Office (SPHSU13), North Central London Research Consortium and the Primary Care Research Network.

## Disclosures

None.

## Supplementary Material

## Novelty and Significance

### What Is New?

- Although black Caribbean and white men had higher pulse wave velocity (PWV) at similar BPs than other men, black Caribbean, African and South Asian women had lower PWVs than white women, despite relatively greater body mass index and fat mass.
- Higher PWV (stiffer arteries) were associated with independent effects from larger waists, and racist experiences; greater waist size (at 11–16 years) intriguingly had a protective effect; earlier BPs had no impact.
- Further adjustment for 5-day physical activity (measured in a subsample) showed protective effects on PWV for number of steps/d or time walking at >100 steps/min per day, whereas time sedentary was adverse.

### What Is Relevant?

- As with PWV’s impact on prognosis, these data suggest independent effects on PWV in youth from body size, current BP, ethnicity and racist experiences, and vitally physical activity per day, with little or no role of previous BP.

### Summary

At given BPs, young African- but no longer Caribbean-origin men had lower arterial stiffness (PWV) than white men in Britain; white women also had higher PWVs than women of other ethnic groups. Large waists, higher current BP, and racist experiences were also associated with higher PWV, whereas greater 5-day measures of physical activity were protective.
