# Supplementary material for: Ethnic Differences in and Childhood Influences on Early Adult Pulse Wave Velocity: The Determinants of Adolescent, Now Young Adult, Social Wellbeing, and Health Longitudinal Study
Source: Hypertension. 2016 May 11;67(6):1133–41. doi: 10.1161/HYPERTENSIONAHA.115.07079 (PMC4861702; doi:10.1161/HYPERTENSIONAHA.115.07079)
Supplement: Supplementary file 1 [file hyp-67-1133-s001.docx]

**Online Supplement**

**Ethnic differences in and childhood influences on early adult PWV: the DASH longitudinal study**

J Kennedy Cruickshank^1^, Maria J Silva^1^, Oarabile R Molaodi^2^, Zinat E Enayat^1^, Aidan Cassidy^2^, Alexis Karamanos^1^, Ursula M Read^2^, Luca Faconti^1^, Philippa Dall^3^, Ben Stansfield^3^, Seeromanie Harding^1^**.**

^1.^Cardiovascular Medicine group, Division of Diabetes & Nutritional Sciences, Kings College London, UK;

^2^Social and Public Health Sciences Unit, Institute of Health and Wellbeing, University of Glasgow, UK;

^3^Institute for Applied Health Research, *Glasgow Caledonian University, Glasgow, UK*

Correspondence: [kennedy.cruickshank@kcl.ac.uk](mailto:kennedy.cruickshank@kcl.ac.uk) ; +44-7588-111187 or

+44-2078484419

Table S1. Descriptive profile of DASH longitudinal pilot follow-up sample: mean (95% CI) or percentage (95% CI) by ethnicity.

| Variables | | White UK | | Black Caribbean | | Black African | | Indian | | Pakistani | | Other | |
| --- | --- | --- | --- | --- | --- | --- | --- | --- | --- | --- | --- | --- | --- |
|  |  | (n = 107) | | (n = 102) | | (n = 132) | | (n = 99) | | Bangladeshi  (n = 111) | | (n = 115) | |
| **21-23y** | | | | | | | | | | | | |  |
| Systolic BP, mmHg | 115.9 (113.4,118.5) | | 114.5 (112.3,116.7) | | 114.2 (112.2,116.1) | | 112.9 (110.6,115.2) | | 111.6 (109.6,113.7) | | 112.8 (110.8,114.8) | |  |
| Diastolic BP, mmHg | 73.17 (71.61,74.72) | | 73.65 (72.11,75.19) | | 72.93 (71.64,74.21) | | 72.52 (70.81,74.22) | | 71.90 (70.44,73.36) | | 70.67 (69.29,72.08) | |  |
| PWV, m/s | 7.3 (6.9,7.6) | | 7.2 (6.8,7.7) | | 6.7 (6.5,7.0) | | 7.1 (6.8,7.4) | | 7.1 (6.8,7.4) | | 7.1 (6.8,7.4) | |  |
| Brachial BP, mmHg | 120.5 (117.7,123.2) | | 119.7 (117.2,122.3) | | 118.7 (116.6,120.9) | | 115.9 (113.5,118.3) | | 115.1 (112.9,117.2) | | 115.2 (113.2,117.1) | |  |
| Height, m | 1.72 (1.71,1.74) | | 1.71 (1.69,1.72) | | 1.71 (1.69,1.73) | | 1.67 (1.65,1.69) | | 1.67 (1.65,1.69) | | 1.69 (1.67,1.71) | |  |
| Waist to height ratio | 0.47 (0.46,0.49) | | 0.49 (0.47,0.50) | | 0.49 (0.47,0.50) | | 0.49 (0.47,0.51) | | 0.49 (0.48,0.51) | | 0.48 (0.47,0.49) | |  |
| BMI, kg/m^†^ | 23.9 (23.0,24.8) | | 26.1 (25.0,27.2) | | 25.9 (25.0,26.7) | | 23.8 (22.8,24.9) | | 24.4 (23.5,25.2) | | 24.5 (23.8,25.3) | |  |
| Overweight status^*,†^ |  | |  | |  | |  | |  | |  | |  |
| Normal Weight | 67.3 (57.8,75.5) | | 50.0 (40.4,59.6) | | 56.8 (48.2,65.0) | | 63.6 (53.7,72.6) | | 64.0 (54.6,72.4) | | 65.2 (56.0,73.4) | |  |
| Overweight | 24.3 (17.1,33.4) | | 28.4 (20.5,38.0) | | 23.5 (17.0,31.5) | | 24.2 (16.8,33.7) | | 25.2 (18.0,34.2) | | 24.4 (17.3,33.1) | |  |
| Obese | 8.4 (4.4,15.4) | | 21.6 (14.6,30.7) | | 19.7 (13.7,27.4) | | 12.1 (7.0,20.2) | | 10.8 (6.2,18.1) | | 10.4 (6.0,17.5) | |  |
|  | | | | | | | | | | | | |  |
| **14-16 y** | | | | | | | | | | | | |  |
| Systolic BP, mmHg | 111.6 (109.2,114.0) | | 111.9 (109.8,114.0) | | 112.2 (110.2,114.2) | | 109.7 (107.5,111.9) | | 110.0 (108.2,111.9) | | 109.0 (106.0,112.1) | |  |
| Diastolic BP, mmHg | 67.2 (65.93) | | 69.8 (68.2,71.3) | | 69.0 (67.9,70.1) | | 69.1 (67.6,70.6) | | 69.1 (67.6,70.6) | | 68.1 (66.2,69.9) | |  |
| Height, m | 1.68 (1.67,1.70) | | 1.67 (1.65,1.69) | | 1.68 (1.66,1.69) | | 1.62 (1.60,1.64) | | 1.63 (1.62,1.65) | | 1.66 (1.64,1.68) | |  |
| Waist to height ratio | 0.42 (0.41,0.43) | | 0.44 (0.43,0.45) | | 0.44 (0.43,0.45) | | 0.43 (0.41,0.44) | | 0.43 (0.42,0.44) | | 0.44 (0.42,0.46) | |  |
| BMI, kg/m^†^ | 20.5 (19.8,21.1) | | 22.7 (21.7,23.7) | | 22.6 (21.9,23.3) | | 20.0 (19.1,20.9) | | 20.1 (19.5,20.8) | | 22.0 (20.9,23.2) | |  |
| Overweight status^*,†^ |  | |  | |  | |  | |  | |  | |  |
| Normal Weight | 87.9 (79.4,93.2) | | 67.4 (57.3,76.1) | | 66.9 (58.1,74.7) | | 80.2 (70.7,87.2) | | 84.7 (76.1,90.1) | | 72.3 (57.8,83.3) | |  |
| Overweight | 8.8 (4.4,16.7) | | 22.1 (14.8,31.6) | | 24.2 (17.4,32.6) | | 15.4 (9.3,24.4) | | 13.3 (7.8,21.6) | | 21.3 (11.8,35.4) | |  |
| Obese | 3.3 (1.1,9.8) | | 10.5 (5.7,1.9) | | 8.9 (5.0,15.4) | | 4.4 (1.6,11.2) | | 2.0 (0.5,7.9) | | 6.4 (2.0,18.2) | |  |
|  | | | | | | | | | | | | |  |
| **11-13 y** | | | | | | | | | | | | |  |
| Systolic BP, mmHg | 109.3 (107.4,111.2) | | 108.6 (106.4,110.7) | | 109.1 (107.5,110.7) | | 107.8 (106.0,109.7) | | 106.4 (104.6,108.3) | | 107.6 (105.8,109.4) | |  |
| Diastolic BP, mmHg | 66.5 (65.1,67.9) | | 67.0 (65.3,68.7) | | 66.5 (65.4,67.6) | | 67.4 (65.8,69.0) | | 66.6 (65.2,67.9) | | 64.7 (63.4,66.0) | |  |
| Height, m | 1.56 (1.54,1.57) | | 1.58 (1.56,1.60) | | 1.59 (1.58,1.60) | | 1.51 (1.49,1.53) | | 1.53 (1.51,1.54) | | 1.55 (1.53,1.57) | |  |
| Waist to height ratio | 0.43 (0.42,0.44) | | 0.43 (0.42,0.44) | | 0.43 (0.42,0.44) | | 0.44 (0.42,0.45) | | 0.43 (0.42,0.44) | | 0.43 (0.42,0.44) | |  |
| BMI, kg/m^†^ | 19.7 (19.0,20.4) | | 21.6 (20.7,22.5) | | 21.5 (20.8,22.2) | | 20.1 (18.7,21.4) | | 19.3 (18.7,20.0) | | 21.6 (19.3,23.9) | |  |
| Overweight status^*,†^ |  | |  | |  | |  | |  | |  | |  |
| Normal Weight | 82.2 (73.8,88.4) | | 58.8 (49.0,68.0) | | 60.6 (52.0,68.6) | | 72.7 (63.1,80.6) | | 81.1 (72.7,87.4) | | 67.5 (58.4,75.5) | |  |
| Overweight | 12.2 (7.2,19.9) | | 28.4 (20.5,38.0) | | 30.3 (23.0,38.7) | | 20.2 (13.4,29.3) | | 12.6 (7.6,20.2) | | 27.2 (19.8,36.1) | |  |
| Obese | 5.6 (2.5,12.0) | | 12.8 (7.5,20.8) | | 9.1 (5.2,15.4) | | 7.1 (3.4,14.2) | | 6.3 (3.0,12.7) | | 5.3 (2.4,11.3) | |  |

Values are mean (95% CI) or ^*^percentage (95% CI).

^†^Weight status: normal weight: <25 kg/m^2^, overweight: >=25 kg/m^2^ and obese: >=30 kg/m^2^.

S2. Final Stata Model to answer comment from Reviewer 1.

log using "L:\PWV Paper\Model.log", replace

clear

use "L:\Dietary Papers\DietaryMaria.dta", clear

/* PWV ~ gender + age + ethnicity + brsBP + WHtR + racism + education + employment at 21-23y */

xi: regress pwv123 i.w1sex w3fs_CAPIage i.ethnicity brsbp123 w3fs_whtr w3fs_racism i.w3fs_educ2 i.w3employment

/* Comment from reviewer: "The complex relationship between BMI components, BP and later PWV could be better represented by changes in the growth trajectory rather than by adjustment for the individual time points of assessment." */

/* add variables from 11-16y - changes in the growth trajectory */

*** BP

generate sbp_1116 = .

replace sbp_1116 = 0 if w2msbpmean>sbp1 & sbp1!=. & w2msbpmean!=. /* increase */

replace sbp_1116 = 1 if w2msbpmean<sbp1 & sbp1!=. & w2msbpmean!=. /* decrease */

replace sbp_1116 = 2 if w2msbpmean==sbp1 & sbp1!=. & w2msbpmean!=. /* remain */

xi: regress pwv123 i.sbp_1116

*** WHtR

generate whtr_1116 = .

replace whtr_1116 = 0 if w2waistheight>w1waistheight & w1waistheight!=. & w2waistheight!=. /* increase */

replace whtr_1116 = 1 if w2waistheight<w1waistheight & w1waistheight!=. & w2waistheight!=. /* decrease */

replace whtr_1116 = 2 if w2waistheight==w1waistheight & w1waistheight!=. & w2waistheight!=. /* remain */

xi: regress pwv123 i.whtr_1116

*** BMI

generate bmi_1116 = .

replace bmi_1116 = 0 if w2mNewbmi>w1bmi & w1bmi!=. & w2mNewbmi!=. /* increase */

replace bmi_1116 = 1 if w2mNewbmi<w1bmi & w1bmi!=. & w2mNewbmi!=. /* decrease */

replace bmi_1116 = 2 if w2mNewbmi==w1bmi & w1bmi!=. & w2mNewbmi!=. /* remain */

xi: regress pwv123 i.bmi_1116

/* Model: PWV ~ gender + age + ethnicity + brsBP + WHtR + racism + education + employment at 21-23y + changes in BP 11-16 + changes in WHtR 11-16 */

xi: regress pwv123 i.w1sex w3fs_CAPIage i.ethnicity brsbp123 w3fs_whtr w3fs_racism i.w3fs_educ2 i.w3employment i.sbp_1116 i.whtr_1116

/* Model: PWV ~ gender + age + ethnicity + brsBP + WHtR + racism + education + employment at 21-23y + changes in BP 11-16 + changes in BMI 11-16 */

xi: regress pwv123 i.w1sex w3fs_CAPIage i.ethnicity brsbp123 w3fs_whtr w3fs_racism i.w3fs_educ2 i.w3employment i.sbp_1116 i.bmi_1116

log close

**. /* PWV ~ gender + age + ethnicity + brsBP + WHtR + racism + education + employment at 21-23y */**

. xi: regress pwv123 i.w1sex w3fs_CAPIage i.ethnicity brsbp123 w3fs_whtr w3fs_racism i.w3fs_educ2 i.w3employment

i.w1sex _Iw1sex_1-2 (naturally coded; _Iw1sex_1 omitted)

i.ethnicity _Iethnicity_0-5 (naturally coded; _Iethnicity_0 omitted)

i.w3fs_educ2 _Iw3fs_educ_0-1 (naturally coded; _Iw3fs_educ_0 omitted)

i.w3employment _Iw3employm_1-2 (naturally coded; _Iw3employm_1 omitted)

Source | SS df MS Number of obs = 456

-------------+------------------------------ F( 12, 443) = 5.64

Model | 132.810282 12 11.0675235 Prob > F = 0.0000

Residual | 869.905149 443 1.96366851 R-squared = 0.1325

-------------+------------------------------ Adj R-squared = 0.1090

Total | 1002.71543 455 2.20377018 Root MSE = 1.4013

-------------------------------------------------------------------------------

pwv123 | Coef. Std. Err. t P>|t| [95% Conf. Interval]

--------------+----------------------------------------------------------------

_Iw1sex_2 | -.4609962 .1488725 -3.10 0.002 -.7535804 -.1684121

w3fs_CAPIage | .1557622 .0925677 1.68 0.093 -.0261641 .3376885

_Iethnicity_1 | -.2561215 .2349252 -1.09 0.276 -.7178279 .2055849

_Iethnicity_2 | -.6099323 .2241848 -2.72 0.007 -1.05053 -.1693344

_Iethnicity_3 | -.2847707 .2352753 -1.21 0.227 -.7471652 .1776237

_Iethnicity_4 | -.3518933 .2377974 -1.48 0.140 -.8192445 .1154579

_Iethnicity_5 | -.4138826 .2273904 -1.82 0.069 -.8607806 .0330154

brsbp123 | .0186041 .0064585 2.88 0.004 .005911 .0312973

w3fs_whtr | 2.747083 1.123246 2.45 0.015 .5395306 4.954634

w3fs_racism | .2461796 .1359573 1.81 0.071 -.0210219 .5133811

_Iw3fs_educ_1 | .0422943 .1388938 0.30 0.761 -.2306784 .315267

_Iw3employm_2 | -.0457959 .1419902 -0.32 0.747 -.3248539 .2332621

_cons | .4475393 2.206153 0.20 0.839 -3.888286 4.783365

-------------------------------------------------------------------------------

**. /* "The complex relationship between BMI components, BP and later PWV could be better represented by changes in the growth trajectory rather than by adjustment for the individual time points of assessment." */**

**. /* add variables from 11-16y - changes in the growth trajectory */**

**. *** BP**

. generate sbp_1116 = .

(665 missing values generated)

. replace sbp_1116 = 0 if w2msbpmean>sbp1 & sbp1!=. & w2msbpmean!=. /* increase */

(320 real changes made)

. replace sbp_1116 = 1 if w2msbpmean<sbp1 & sbp1!=. & w2msbpmean!=. /* decrease */

(213 real changes made)

. replace sbp_1116 = 2 if w2msbpmean==sbp1 & sbp1!=. & w2msbpmean!=. /* remain */

(9 real changes made)

. xi: regress pwv123 i.sbp_1116

i.sbp_1116 _Isbp_1116_0-2 (naturally coded; _Isbp_1116_0 omitted)

Source | SS df MS Number of obs = 456

-------------+------------------------------ F( 2, 453) = 0.27

Model | 1.31967821 2 .659839107 Prob > F = 0.7608

Residual | 1092.7979 453 2.41235739 R-squared = 0.0012

-------------+------------------------------ Adj R-squared = -0.0032

Total | 1094.11757 455 2.40465401 Root MSE = 1.5532

------------------------------------------------------------------------------

pwv123 | Coef. Std. Err. t P>|t| [95% Conf. Interval]

-------------+----------------------------------------------------------------

_Isbp_1116_1 | -.104801 .1502125 -0.70 0.486 -.4000008 .1903988

_Isbp_1116_2 | -.1698889 .5262835 -0.32 0.747 -1.204149 .8643712

_cons | 7.106741 .0945233 75.19 0.000 6.920982 7.292499

------------------------------------------------------------------------------

**. *** WHtR**

. generate whtr_1116 = .

(665 missing values generated)

. replace whtr_1116 = 0 if w2waistheight>w1waistheight & w1waistheight!=. & w2waistheigh

> t!=. /* increase */

(297 real changes made)

. replace whtr_1116 = 1 if w2waistheight<w1waistheight & w1waistheight!=. & w2waistheigh

> t!=. /* decrease */

(240 real changes made)

. replace whtr_1116 = 2 if w2waistheight==w1waistheight & w1waistheight!=. & w2waistheig

> ht!=. /* remain */

(0 real changes made)

. xi: regress pwv123 i.whtr_1116

i.whtr_1116 _Iwhtr_1116_0-1 (naturally coded; _Iwhtr_1116_0 omitted)

Source | SS df MS Number of obs = 454

-------------+------------------------------ F( 1, 452) = 0.73

Model | 1.73794116 1 1.73794116 Prob > F = 0.3930

Residual | 1074.59491 452 2.37742236 R-squared = 0.0016

-------------+------------------------------ Adj R-squared = -0.0006

Total | 1076.33285 453 2.3760107 Root MSE = 1.5419

-------------------------------------------------------------------------------

pwv123 | Coef. Std. Err. t P>|t| [95% Conf. Interval]

--------------+----------------------------------------------------------------

_Iwhtr_1116_1 | .1241785 .1452385 0.85 0.393 -.1612481 .409605

_cons | 6.995108 .0983073 71.16 0.000 6.801912 7.188304

-------------------------------------------------------------------------------

**. *** BMI**

. generate bmi_1116 = .

(665 missing values generated)

. replace bmi_1116 = 0 if w2mNewbmi>w1bmi & w1bmi!=. & w2mNewbmi!=. /* increase */

(387 real changes made)

. replace bmi_1116 = 1 if w2mNewbmi<w1bmi & w1bmi!=. & w2mNewbmi!=. /* decrease */

(147 real changes made)

. replace bmi_1116 = 2 if w2mNewbmi==w1bmi & w1bmi!=. & w2mNewbmi!=. /* remain */

(0 real changes made)

. xi: regress pwv123 i.bmi_1116

i.bmi_1116 _Ibmi_1116_0-1 (naturally coded; _Ibmi_1116_0 omitted)

Source | SS df MS Number of obs = 451

-------------+------------------------------ F( 1, 449) = 0.43

Model | 1.02508687 1 1.02508687 Prob > F = 0.5141

Residual | 1079.40809 449 2.40402693 R-squared = 0.0009

-------------+------------------------------ Adj R-squared = -0.0013

Total | 1080.43318 450 2.40096261 Root MSE = 1.5505

------------------------------------------------------------------------------

pwv123 | Coef. Std. Err. t P>|t| [95% Conf. Interval]

-------------+----------------------------------------------------------------

_Ibmi_1116_1 | .1050166 .1608227 0.65 0.514 -.211042 .4210752

_cons | 7.022719 .0866752 81.02 0.000 6.852379 7.193058

------------------------------------------------------------------------------

**. /* Model: PWV ~ gender + age + ethnicity + brachial BP + WHtR + racism + education + employment at 21-23y + changes in BP 11-16 + changes in WHtR 11-16 */**

. xi: regress pwv123 i.w1sex w3fs_CAPIage i.ethnicity brsbp123 w3fs_whtr w3fs_racism i.w3fs_educ2 i.w3employment i.sbp_1116 i.whtr_1116

i.w1sex _Iw1sex_1-2 (naturally coded; _Iw1sex_1 omitted)

i.ethnicity _Iethnicity_0-5 (naturally coded; _Iethnicity_0 omitted)

i.w3fs_educ2 _Iw3fs_educ_0-1 (naturally coded; _Iw3fs_educ_0 omitted)

i.w3employment _Iw3employm_1-2 (naturally coded; _Iw3employm_1 omitted)

i.sbp_1116 _Isbp_1116_0-2 (naturally coded; _Isbp_1116_0 omitted)

i.whtr_1116 _Iwhtr_1116_0-1 (naturally coded; _Iwhtr_1116_0 omitted)

Source | SS df MS Number of obs = 366

-------------+------------------------------ F( 15, 350) = 3.87

Model | 127.204164 15 8.48027759 Prob > F = 0.0000

Residual | 767.293937 350 2.19226839 R-squared = 0.1422

-------------+------------------------------ Adj R-squared = 0.1054

Total | 894.498101 365 2.45067973 Root MSE = 1.4806

-------------------------------------------------------------------------------

pwv123 | Coef. Std. Err. t P>|t| [95% Conf. Interval]

--------------+----------------------------------------------------------------

_Iw1sex_2 | -.4268304 .1782936 -2.39 0.017 -.7774919 -.0761688

w3fs_CAPIage | .1056651 .1088231 0.97 0.332 -.1083643 .3196946

_Iethnicity_1 | -.453483 .2705435 -1.68 0.095 -.9855785 .0786124

_Iethnicity_2 | -.7244259 .255687 -2.83 0.005 -1.227302 -.2215496

_Iethnicity_3 | -.4263644 .2658141 -1.60 0.110 -.9491583 .0964296

_Iethnicity_4 | -.5045443 .2708266 -1.86 0.063 -1.037197 .028108

_Iethnicity_5 | -.4933645 .3255072 -1.52 0.131 -1.133561 .1468317

brsbp123 | .020238 .007486 2.70 0.007 .0055148 .0349611

w3fs_whtr | 3.364706 1.323302 2.54 0.011 .7620817 5.967331

w3fs_racism | .3250482 .1615561 2.01 0.045 .0073054 .642791

_Iw3fs_educ_1 | .0787567 .1665924 0.47 0.637 -.2488913 .4064047

_Iw3employm_2 | -.0370581 .1680448 -0.22 0.826 -.3675628 .2934465

_Isbp_1116_1 | .0549301 .1673368 0.33 0.743 -.274182 .3840421

_Isbp_1116_2 | -.4455873 .5473641 -0.81 0.416 -1.522124 .6309493

_Iwhtr_1116_1 | -.0011191 .1597021 -0.01 0.994 -.3152156 .3129774

_cons | 1.103922 2.584207 0.43 0.670 -3.978606 6.186449

-------------------------------------------------------------------------------

**. /* Model: PWV ~ gender + age + ethnicity + brsBP + WHtR + racism + education + employment at 21-23y + changes in BP 11-16 + changes in BMI 11-16 */**

. xi: regress pwv123 i.w1sex w3fs_CAPIage i.ethnicity brsbp123 w3fs_whtr w3fs_racism i.w3fs_educ2 i.w3employment i.sbp_1116 i.bmi_1116

i.w1sex _Iw1sex_1-2 (naturally coded; _Iw1sex_1 omitted)

i.ethnicity _Iethnicity_0-5 (naturally coded; _Iethnicity_0 omitted)

i.w3fs_educ2 _Iw3fs_educ_0-1 (naturally coded; _Iw3fs_educ_0 omitted)

i.w3employment _Iw3employm_1-2 (naturally coded; _Iw3employm_1 omitted)

i.sbp_1116 _Isbp_1116_0-2 (naturally coded; _Isbp_1116_0 omitted)

i.bmi_1116 _Ibmi_1116_0-1 (naturally coded; _Ibmi_1116_0 omitted)

Source | SS df MS Number of obs = 362

-------------+------------------------------ F( 15, 346) = 3.76

Model | 123.909888 15 8.2606592 Prob > F = 0.0000

Residual | 760.308305 346 2.19742285 R-squared = 0.1401

-------------+------------------------------ Adj R-squared = 0.1029

Total | 884.218193 361 2.44935788 Root MSE = 1.4824

-------------------------------------------------------------------------------

pwv123 | Coef. Std. Err. t P>|t| [95% Conf. Interval]

--------------+----------------------------------------------------------------

_Iw1sex_2 | -.488093 .1771799 -2.75 0.006 -.8365782 -.1396077

w3fs_CAPIage | .1093935 .1093899 1.00 0.318 -.1057593 .3245463

_Iethnicity_1 | -.3685352 .2733061 -1.35 0.178 -.9060857 .1690152

_Iethnicity_2 | -.6482595 .2580861 -2.51 0.012 -1.155874 -.1406445

_Iethnicity_3 | -.3657899 .2691469 -1.36 0.175 -.8951598 .1635801

_Iethnicity_4 | -.3664036 .2740187 -1.34 0.182 -.9053557 .1725484

_Iethnicity_5 | -.4122746 .3284145 -1.26 0.210 -1.058215 .2336655

brsbp123 | .0190811 .0075619 2.52 0.012 .004208 .0339542

w3fs_whtr | 3.569255 1.339961 2.66 0.008 .9337612 6.204748

w3fs_racism | .2566324 .1627665 1.58 0.116 -.0635039 .5767687

_Iw3fs_educ_1 | .1078661 .1681167 0.64 0.522 -.2227932 .4385253

_Iw3employm_2 | -.0375939 .169103 -0.22 0.824 -.3701931 .2950052

_Isbp_1116_1 | .0554826 .1678668 0.33 0.741 -.2746853 .3856504

_Isbp_1116_2 | -.4385063 .548283 -0.80 0.424 -1.516893 .6398809

_Ibmi_1116_1 | .1827684 .1720003 1.06 0.289 -.1555293 .5210661

_cons | .9805646 2.608008 0.38 0.707 -4.148981 6.11011

-------------------------------------------------------------------------------
